# The Country Doctor

**Published:** 1886-03-20

**Authors:** 


THE COUNTRY DOCTOR.—City practitioners have no conception of the difficulties encountered by the Country Doctor. In the city, the physician can always promptly secure consultation in any case of emergency, and the young physician of the city, from this very fact, is not likely to become so very selfreliant and determined as the young doctor of the country. The manner in which emergencies are met and overcome in the country would surprise the city physician, who has at hand the best counsel and every needed facility. Not long ago a country practitioner related to the editor how he had succeeded in removing an abnormally large fœtus from a woman in labor with an ordinary pocket-knife, and crooked piece of iron obtained from a mowing blade, there being no other instruments at hand, and no chance to secure consultation or aid from any point within twenty miles. The editor of this journal, in a surgical case in the country, could procure no operating table. A country physician, who was present, unhinged a door, which being placed across two chairs, answered our purposes admirably. We are cognizant of another case in which the doctor drew the urine from the bladder of a female with an ordinary reed pipe-stem, rounded off at the point and well greased with suet. In yet another case, the practitioner, in a tedious labor case, having no forceps, succeeded in using sufficient traction by means of the handles of two large spoons.

We would be pleased to have our country friends relate, for publication in the RECORD, any experiences of a similar character which they have had to contend with in country practice.

